# A Varying Coefficient Model to Measure the Effectiveness of Mass Media Anti-Smoking Campaigns in Generating Calls to a Quitline

**DOI:** 10.2188/jea.JE20090105

**Published:** 2010-11-05

**Authors:** Quang M. Bui, Richard M. Huggins, Wen-Han Hwang, Victoria White, Bircan Erbas

**Affiliations:** 1 Centre for Molecular, Environmental, Genetic & Analytic Epidemiology, The University of Melbourne, Victoria, Australia; 2 Department of Mathematics and Statistics, The University of Melbourne, Victoria, Australia; 3 Department of Applied Mathematics, National Chung Hsin University, Taichung, Taiwan; 4 The Cancer Council Victoria; 5 School of Public Health, La Trobe University, Victoria, Australia

**Keywords:** regression models, semi-parametric, smoking cessation program

## Abstract

**Background:**

Anti-smoking advertisements are an effective population-based smoking reduction strategy. The Quitline telephone service provides a first point of contact for adults considering quitting. Because of data complexity, the relationship between anti-smoking advertising placement, intensity, and time trends in total call volume is poorly understood. In this study we use a recently developed semi-varying coefficient model to elucidate this relationship.

**Methods:**

Semi-varying coefficient models comprise parametric and nonparametric components. The model is fitted to the daily number of calls to Quitline in Victoria, Australia to estimate a nonparametric long-term trend and parametric terms for day-of-the-week effects and to clarify the relationship with target audience rating points (TARPs) for the Quit and nicotine replacement advertising campaigns.

**Results:**

The number of calls to Quitline increased with the TARP value of both the Quit and other smoking cessation advertisement; the TARP values associated with the Quit program were almost twice as effective. The varying coefficient term was statistically significant for peak periods with little or no advertising.

**Conclusions:**

Semi-varying coefficient models are useful for modeling public health data when there is little or no information on other factors related to the at-risk population. These models are well suited to modeling call volume to Quitline, because the varying coefficient allowed the underlying time trend to depend on fixed covariates that also vary with time, thereby explaining more of the variation in the call model.

## INTRODUCTION

Smoking is the single largest preventable cause of death and disease in Australia. It was estimated that over 19 000 people died from tobacco-related diseases in 1998.^1^ The total social costs of tobacco use have been estimated at over 21 billion Australian dollars annually—including health care, hospitalizations, loss of productivity and earnings due to premature death, and other direct and indirect costs.^2^ Thus, encouraging individuals to stop smoking is an important public health challenge.

In Australia the prevalence of smoking consistently declined from the early 1960s to the early 1990s, but stalled in the mid-1990s.^3^^,^^4^ In an effort to reduce smoking prevalence, in June 1997, the Australian federal government collaborated with the Australian States and Territories to launch the National Tobacco Campaign (NTC). This is Australia’s most intense and sustained mass media tobacco control campaign. The major aim of this initiative was to show television commercials with intense content, so that smokers would immediately attempt to quit. The target for the NTC campaigns was smokers between 18 and 40 years of age. The Quitline telephone helpline service is a population telephone-based program that was promoted as part of the NTC program and provides a first point of contact to assist smokers who wish to quit. It is a flexible and cost-effective campaign, and is easily accessible to a large population.^5^ It has also been shown to be an effective aid in smoking cessation.^6^^–^^8^

**Figure 1.**
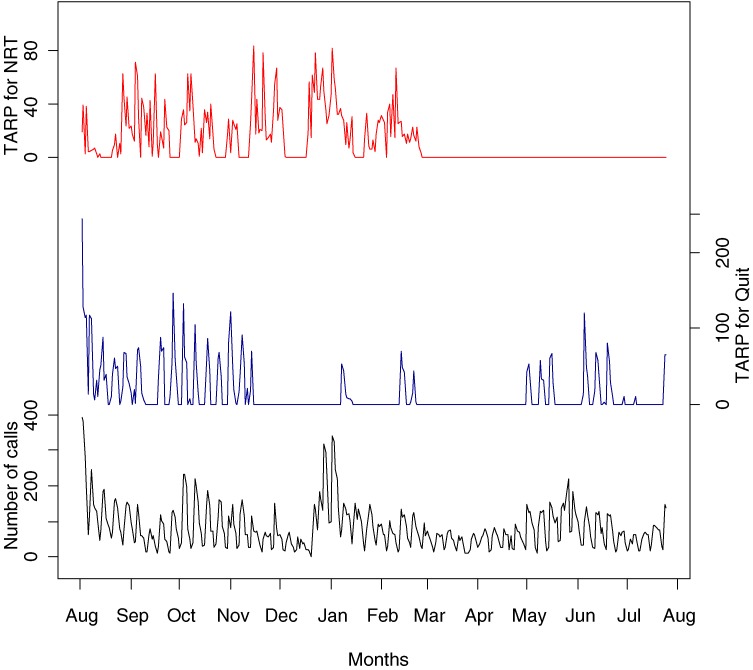
Number of calls to Quitline (bottom), TARPs for the Quit program (middle), and TARPs for the nicotine replacement therapy (NRT) program (top) from 6 August 2000 through 30 July 2001.

Several studies have examined the association between the amount of mass media anti-smoking advertising, as measured by target audience rating points (TARPs), and number of calls to a telephone-based quitline.^8^^–^^10^ TARPs are a measure of television advertising weight, and are used to indicate the number of people in a particular demographic group exposed to an advertisement within a specified period of time.^17^ Miller et al^8^ used relatively simple regression analysis to show that there is a linear relationship between number of calls and TARPs. Their method assumes that the relationship was fixed through time. However, this assumption may not be valid as these relationships may vary over time in response to changes in the type, intensity, and placement of advertisements. In addition, the number of calls may not depend entirely on TARPs, but also on other, unknown factors. For example, in Figure 1 we plot the daily number of calls (bottom) to Quitline in Victoria, Australia, from August 2000 until the end of July 2001, along with 2 anti-smoking advertising campaign variables: TARPs for the Quit campaign (middle) and the nicotine replacement therapy (NRT) campaign (top) in Victoria. The NRT campaigns were run by pharmaceutical companies promoting their products, eg, patches, gum, and inhalers. The figure suggests an increase in number of calls when the TARPs for both campaigns increase, although this increase did not always correspond with the size of the TARPs, as would have been expected, particularly from mid-December 2000 to mid-January 2001, which is the Australian summer holiday period.

Figure 1 also shows that, in 2001, the TARPs for both campaigns were 0 from March to May, and from mid-May to early June, and the TARPs were lowest from mid-June until approximately the end of August.

**Figure 2.**
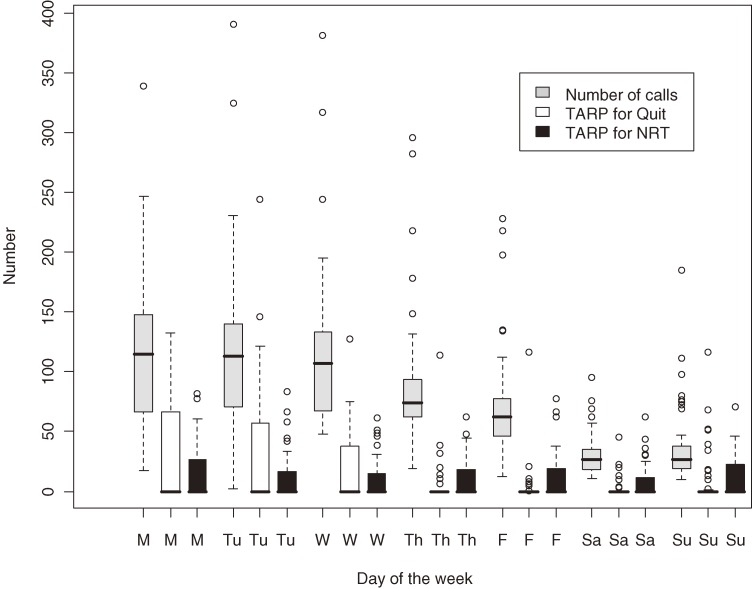
Box-and-whisker plots of number of calls to Quitline and TARPs for the Quit and nicotine replacement therapy (NRT) programs by day of the week.

Figure 2 shows box-and-whisker plots of the daily number of calls and TARPs for both campaigns for each day of the week of the study period. The number of calls shows a weekly cyclic pattern, with a peak from Monday through Wednesday and then a gradual decline to a minimum on Sunday. However, TARPs by day behaves differently for each campaign: TARPs for Quit are centered on the period from Monday through Wednesday, similar to the trend in call volume; however, TARPs for the NRT campaign are more evenly spread throughout the week. Moreover, the number of calls are relatively high on Thursday and Friday, when the TARPs for Quit are low.

To model a complex process that evolves over time, as in the present case, standard regression models can be replaced by a number of models, such as generalized additive models^11^ and semi-varying coefficient models.^12^ In this study we sought to model the relationship between number of calls to Quitline Victoria, TARPs for both the Quit and NRT campaigns, and day-of-the-week effects, using a semi-varying coefficient model comprising parametric and nonparametric components whose coefficients are allowed to change smoothly with time. By doing so, we were able to evaluate the usefulness of this method in modeling complex public health data.

## METHODS

### Data

We use daily data on total number of calls to Quitline Victoria, total number of Quit antismoking advertisements on free-to-air media, and TARPs for both the Quit and NRT programs during the period from 6 August 2000 through 31 July 2001. The data used in this study were de-identified and collapsed into daily total counts for the purposes of the study. Therefore, the use of the data did not require ethical approval.

### Statistical methods

Semiparametric models allow us to fit parametric models to known features of the data and nonparametric terms to the unknown component. This aids both in making inferences about the known features and in detecting unknown structures. For the purpose of this study, there are 3 types of individuals: smokers who are not considering quitting, smokers that are considering calling Quitline and quitting, and smokers that have called Quitline. Suppose observations are taken over the time interval $\left(t_{j-1},t_{j}\right]$, j=1,…,J, $0=t_{0}\leq \cdots \leq t_{n}=\tau$. In our context, this time interval is defined as a day. At the start of day *j* there are *N_j_* people “at risk” of giving up smoking. The probability they call Quitline on that day is *p_j_*. In general, *N_j_* and *p_j_* are not known. Let *Y_j_* be the number of these at-risk individuals that do call Quitline on day *j*; therefore, there are *N_j_* − *Y_j_* individuals remaining, consisting of individuals who do not give up smoking and those who are thinking of calling Quitline. Let *N_j_*_+1_ = *N_j_* − *Y_j_* be the number of individuals at risk the next day, and let *F_j_* be the number of calls for each day on the series up to and including day *j*, ie, $F_{j}=\{Y_{0},Y_{1},\ldots ,Y_{j}\}$. Consider a chain binomial model where the conditional distribution $Y_{j}|F_{j-1}\sim bin(N_{j},p_{j})$ and the number at risk at the start of the *j*th interval is *N_j_* = *N_j_*_−1_ − *Y_j_*_−1_, with *N*_0_ being the number initially at risk. If the *N_j_* are so large that *N_j_*/*N_j_*_−1_ ≈ 1, then

$$E(Y_{j}|F_{j-1})=N_{j}p_{j}={\alpha^{\prime }}_{j}N_{j}Y_{j-1}/N_{j-1}\approx Y_{j-1}{\alpha^{\prime }}_{j},$$

where ${\alpha^{\prime }}_{j}=p_{j}N_{j-1}Y_{j-1}\approx p_{j}p_{j-1}$. If *p_j_* are small, then 1 − *p_j_* ≈ 1 and thus

$$Var(Y_{j}|F_{j-1})=N_{j}p_{j}(1-p_{j})\approx N_{j}p_{j}\approx Y_{j-1}{\alpha^{\prime }}_{j},$$

so it is reasonable to assume $Y_{j}|F_{j-1}\sim \mathrm{Poisson}(Y_{j-1}{\alpha^{\prime }}_{j})$. This model is a type of first-order autoregressive conditional Poisson time series with varying coefficients ${\alpha^{\prime }}_{j}$. We extend this model by parameterizing the coefficients as ${\alpha^{\prime }}_{j}=\alpha_{j}+X_{j}^{T}\beta$, where $X_{j}^{T}=(X_{j1},X_{j2},\ldots ,X_{jp})$ is a row vector of covariates of length *p* and $\beta ={\left(\beta_{1},\beta_{2},\ldots ,\beta_{p}\right)}^{T}$ is the column vector of parameters of the same length. The term $X_{j}^{T}\beta$ allows us to examine the effect of covariates on the at-risk population. There is also an immigration component to the at-risk population, and some smokers will call Quitline without entering an at-risk phase, and other individuals become at risk. The numbers of the latter are not observed. We assume the number of individuals that were previously not at risk of quitting that do call Quitline on day *j* has a Poisson distribution with mean $Z_{j}^{T}\gamma$, where $Z_{j}^{T}=(Z_{j1},Z_{j2},\ldots ,Z_{jq})$ is a row vector of covariates of length *q* and $\gamma ={\left(\gamma_{1},\gamma_{2},\ldots ,\gamma_{p}\right)}^{T}$ is the column vector of parameters of the same length. Thus, our model for the mean number of calls to Quitline on each day is $E(Y_{j}|F_{j-1})=Y_{j-1}(\alpha_{j}+X_{j}^{T}\beta )+Z_{j}^{T}\gamma$.

The first term of the model is *Y_j_*_−1_*α_j_*, where *α_j_* represents the nonparametric parts of the model. An examination of the estimated *α_j_* allows us to detect previously unknown structures in the data. The term $Y_{j-1}X_{j}^{T}\beta$ reflects the effects of the covariates on the at-risk cohort. The remaining term $Z_{j}^{T}\gamma$ represents the mean number of individuals that immigrate into the at-risk population then immediately call Quitline. Other immigrants who do not call Quitline are absorbed into the cohort at risk of calling the next day.

Clearly, more complex models are possible, but the present model can be fitted to the data, and the nonparametric component allows us to find structures that are not included in the parametric components.

We compared the performance of our model with 4 other models (see Table 1) using the mean square error (MSE), ie, $\mathrm{MSE}=(1/J)\sum_{j=1}^{J}{\left(Y_{j}-{\hat{Y}}_{j}\right)}^{2}$, where ${\hat{Y}}_{j}$ is the fitted value. Model 1 is a linear regression model used by Miller et al.^8^ Model 2 is the same as model 1, but assumes a Poisson distribution for the outcome variable. Model 3 is similar to our model (Model 5), but the varying coefficient *α_j_* is replaced by the constant coefficient *α*. Model 4 is the model used by Richard et al.^12^

**Table 1.** Mean square error for 5 models for the number of calls to the Quitline and NRT campaigns

Mean model	Distribution of response variable *Y_j_*	Mean square error (MSE)
Model 1: $E(Y_{j})=\alpha +\gamma_{7}TARP_{j}^{Quit}+\gamma_{8}TARP_{j}^{NRT}$	Gaussian	2348
Model 2: $E(Y_{j})=\alpha +\gamma_{7}TARP_{j}^{Quit}+\gamma_{8}TARP_{j}^{NRT}$	Poisson	2348
Model 3: $E(Y_{j}|F_{j-1})=Y_{j-1}(\alpha +{\text{X}}_{j}^{T}\beta )+{\text{Z}}_{j}^{T}\gamma$	Conditional Poisson	915
Model 4: $E(Y_{j}|F_{j-1})=Y_{j-1}\alpha_{j}+{\text{Z}}_{j}^{T}\gamma$	Conditional Poisson	863
Model 5: $E(Y_{j}|F_{j-1})=Y_{j-1}(\alpha_{j}+X_{j}^{T}\beta )+Z_{j}^{T}\gamma$	Conditional Poisson	820

Abbreviation: NRT, nicotine replacement therapy.

### Standard errors and goodness of fit

To apply the model to the Quit data, we let $X_{j}^{T}=(X_{jMon},X_{jTues},\ldots ,X_{jSat})$ be a vector of covariates of day of the week, where $X_{jMon}$ is the indicator function which equals to 1 for Monday and 0 otherwise, and similarly for $X_{jTues},\ldots ,X_{jSat}$. Note that we set Sunday to 0. The vector **Z***_j_* includes **X***_j_* and the TARPs for both the Quit and NRT campaigns. That is, the model for the mean number of calls is

$$\begin{array}{l} E(Y_{j}|F_{j-1})=\alpha_{j}Y_{j-1}+\beta_{1}X_{jMon}Y_{j-1}+\beta_{2}X_{jTues}Y_{j-1}+\cdots \\ +\beta_{6}X_{jSat}Y_{j-1}+\gamma_{1}X_{jMon}+\gamma_{2}X_{jTues}+\cdots \\ +\gamma_{6}X_{jSat}+\gamma_{7}TARP_{j}^{Quit}+\gamma_{8}TAPR_{j}^{NRT}. \end{array}$$

Parameters in the model were estimated by the method described in Huggins et al.^12^ A closed-form expression of standard errors of the parametric component of the model was not found and must be estimated by the bootstrap method. This can be carried out by considering *Y*_1_ as fixed and by generating *Y_j_* according to $Poisson\{Y_{j-1}({\hat{\alpha }}_{j}+X_{j}^{T}\hat{\beta })+Z_{j}^{T}\hat{\gamma }\}$ distribution. However, this approach could result in reporting an incorrect bootstrap estimate of standard errors of parameters if the data are overdispersed. To adjust for overdispersion, we suggest the use of nonparametric bootstrap as follow:

Let *Var*(*Y_j_*|*F_j_*_−1_) = *ϕμ_j_* and the modified Pearson residual be $r_{j}^{*}=(Y_{j}-{\hat{\mu }}_{j})/\sqrt{\phi {\hat{\mu }}_{j}}$. Solving $Var(r_{j}^{*})-1=0$ to obtain value for *ϕ* and use this value to compute $r_{j}^{*}$. A bootstrap sample is generated by randomly sampling with replacement *J* observations from $r_{j}^{*}$, denoted by $r_{j}^{*b}$, and then calculating estimates *β* and *γ* using $Y_{j}^{b}={\hat{\mu }}_{j}+r_{j}^{*b}\sqrt{\phi {\hat{\mu }}_{j}}$. This procedure is repeated a number of times to obtain a set of bootstrap estimates of parameters. From this set of estimates, the standard error of estimates is estimated by the standard deviation of the bootstrap replicates.

To assess the goodness of fit of the model, we use deviance R-square for Poisson regression models,^13^ which is given by

$$R^{2}=\sum \{Y_{j}\ln({\hat{\mu }}_{j}/\bar{Y})-(Y_{j}-{\hat{\mu }}_{j})\}/\sum Y_{j}\ln(Y_{j}/\bar{Y}).$$

The Poisson model assumes that the conditional mean is equal to the conditional variance. This assumption is not tenable if the data are overdispersed—a consequence of the conditional variance exceeding the conditional mean, and thus resulting in reporting standard errors that are no longer correct. An informal technique for assessing overdispersion is to compute Pearson residuals, ie, $r_{j}=(Y_{j}-{\hat{\mu }}_{j})/\sqrt{{\hat{\mu }}_{j}}$, and use these residuals to check for 0 mean and variance of 1. A more formal approach is to test the Poisson assumption against the negative binomial assumption,^14^ where the conditional variance is of the form *Var*(*Y_j_*|*F_j_*_−1_) = *ϕμ_j_*, where *ϕ* is the dispersion parameter, by performing the following regression $Y_{j}^{*}=\phi +u_{j}$, where $Y_{j}^{*}=[{\left(Y_{j}-{\hat{\mu }}_{j}\right)}^{2}-Y_{j}]/{\hat{\mu }}_{j}$, ${\hat{\mu }}_{j}$ is the estimated conditional mean and *u_j_* is an error term. The parameter *ϕ* is asymptotically normal under the null hypothesis of no dispersion against the alternative of overdispersion of the negative binomial.

All analyses were conducted using our own programs written in R language (version 2.8.1; http://www.R-project.org).

## RESULTS

**Figure 3.**
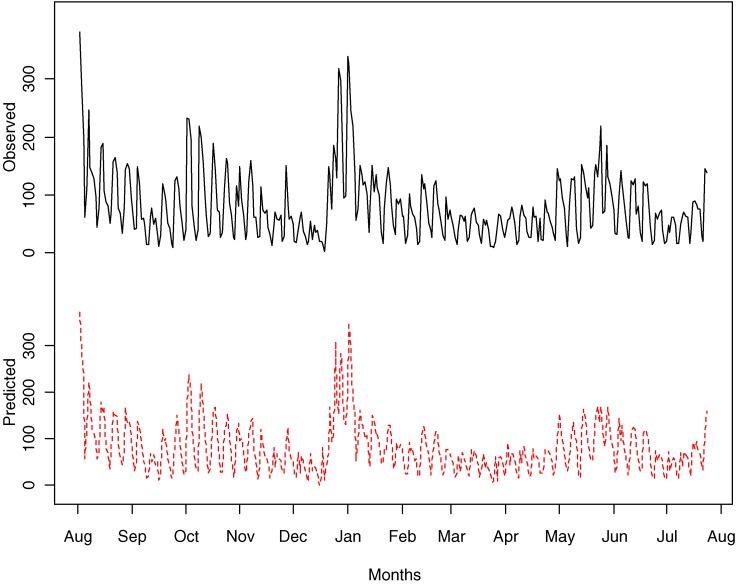
Observed daily total number of calls to Quitline (solid line) and estimated conditional mean (broken line) from fitting a varying coefficient model.

We fit the varying coefficient models to the daily total number of calls to Quitline (outcome variable). The model fit the data reasonably well, with deviance *R*^2^ = 0.839 and estimated conditional mean values closely agreeing with the observed values (Figure 3).

A plot of the estimated density of the Pearson residuals (not shown) showed that most of the residuals were distributed symmetrically around 0. A simple calculation, however, revealed that the sample mean and variance of the Pearson residuals were 0.116 and 6.562, respectively, indicating that the data were overdispersed. The formal regression test for overdispersion gave $\hat{\phi }$ = 5.518 with a *P*-value <0.0001, providing conclusive evidence of overdispersion. Therefore, we employed a nonparametric bootstrap procedure to estimate standard errors of fixed effect parameters. The estimated parametric effects are shown in Table 2. Note that these estimated parameters are all relative to Sunday. In this table, the parameters *β*, together with varying coefficients *α_j_*, represent effects for the total number of calls to Quitline on the previous day, while the parameter *γ* represents the day-of-the-week fixed effects (*γ*_1_ to *γ*_6_), in addition to the effects of the advertisements (*γ*_7_ and *γ*_8_) on that day.

**Table 2.** Estimated fixed parametric effects with bootstrap estimates of standard error based on 5000 bootstrap samples

Parameter	Estimate	s.e.	z	*P*-value
β_1_ (Mon)	0.080	0.217	0.371	0.7108
β_2_ (Tue)	−0.201	0.101	−1.987	0.0469
β_3_ (Wed)	−0.394	0.103	−3.823	0.0001
β_4_ (Thu)	−0.487	0.118	−4.128	<0.0001
β_5_ (Fri)	−0.433	0.131	−3.300	0.0010
β_6_ (Sat)	−0.754	0.117	−6.452	<0.0001

γ_1_ (Mon)	51.09	8.569	5.962	<0.0001
γ_2_ (Tue)	−3.292	9.944	−0.331	0.7406
γ_3_ (Wed)	28.25	10.27	2.751	0.0059
γ_4_ (Thu)	23.29	11.38	2.046	0.0407
γ_5_ (Fri)	14.75	10.03	1.471	0.1412
γ_6_ (Sat)	7.712	6.982	1.105	0.2693

γ_7_ (TARPs Quit)	0.488	0.083	5.855	<0.0001
γ_8_ (TARPs NRT)	0.226	0.099	2.291	0.0220

Abbreviation: NRT, nicotine replacement therapy.

**Figure 4.**
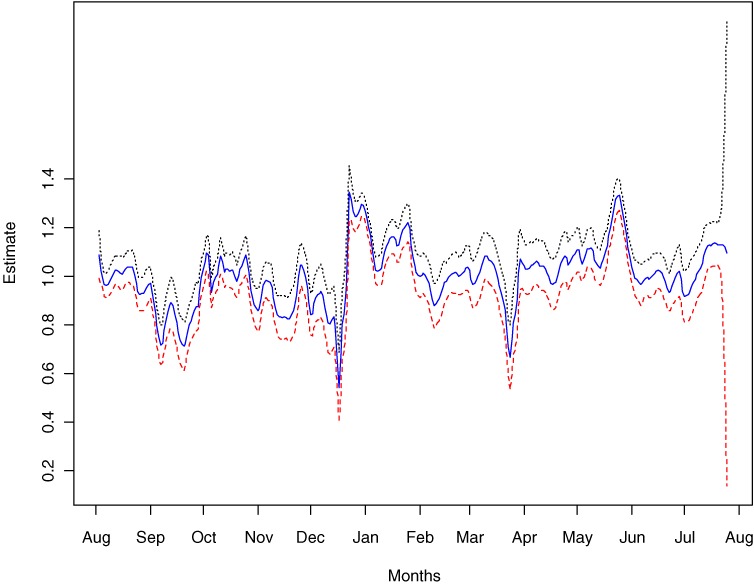
Estimated varying coefficients ${\hat{\alpha }}_{j}$ and their 95% confidence interval.

In Figure 4 we graph the estimated varying coefficients ${\hat{\alpha }}_{j}$ and the nominal 95% confidence interval. The varying coefficients represent the effects not explained by the TARPs for both the Quit and NRT programs.

**Figure 5.**
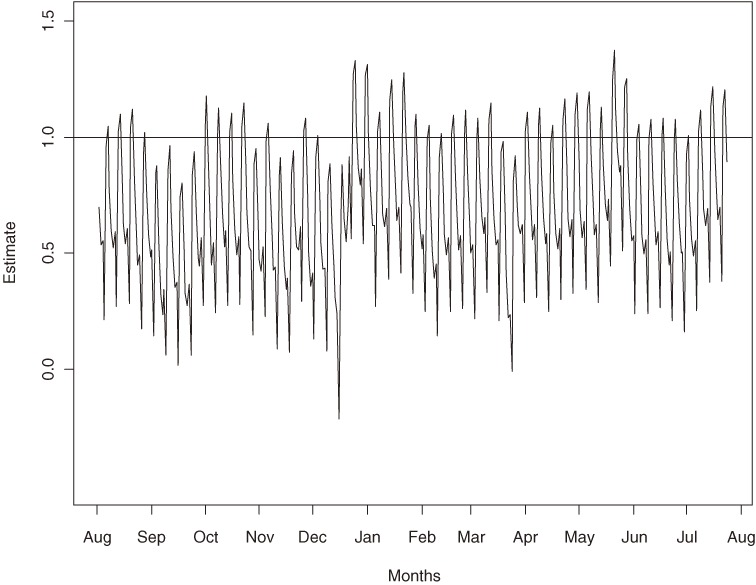
Plot of ${\hat{\alpha }}_{j}+X_{j}^{T}\hat{\beta }$.

The negative estimates of *β*_2_ (Tuesday) to *β*_6_ (Saturday) reflect the effect of a large number of calls on 1 day, leading to a decline in calls the next day. A large number of calls on 1 day reduces the population cohort that can potentially make a call the next day, and vice versa for a positive estimate of *β*_1_ (Monday). These effects are shown in Figure 5, where we plot the combined effect of ${\hat{\alpha }}_{j}$ and fixed parameters ${\hat{\beta }}_{j}$, ie, ${\hat{\alpha }}_{j}+X_{j}^{T}\hat{\beta }$. The figure shows a weekly cyclic pattern, with maximum values on Monday and minimum values on Saturday.

The daily effects represented by the *γ*’s are perhaps more representative of the susceptibility of the at-risk population of smokers to advertisements, and this may be regarded as a combination of their TV viewing habits and psychological state. They do represent the day effect if there were no calls made on the previous day. For example, if there were no calls on a Sunday we would expect about 51 additional calls on a Monday, whereas if there were 100 calls on a Sunday we expect only 59 additional calls. Furthermore, as expected, the number of calls to Quitline increased with the TARP value of either type of advertisement, and the TARPs associated with the Quit program were almost twice as effective.

**Figure 6.**
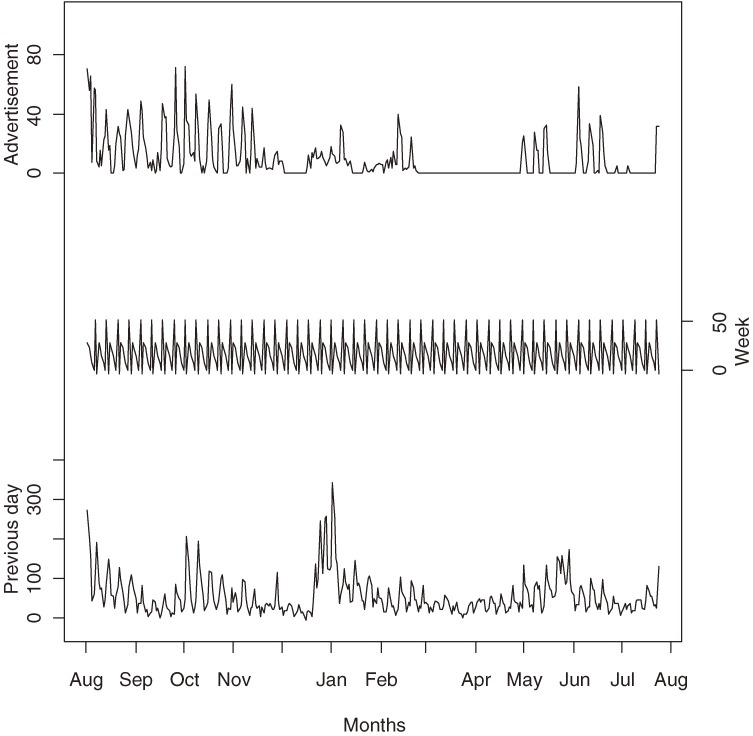
Estimated conditional mean number of calls contributed from previous day effects $Y_{j-1}({\hat{\alpha }}_{j}+X_{j}^{T}\hat{\beta })$ (bottom), day-of-the-week effects $Z_{j}^{T}{\hat{\gamma }}^{a}$ (middle), where ${\hat{\gamma }}^{a}={\left({\hat{\gamma }}_{1},\cdots ,{\hat{\gamma }}_{6}\right)}^{T}$, and advertisement effects $Z_{j}^{T}{\hat{\gamma }}^{b}$ (top), where ${\hat{\gamma }}^{b}={\left({\hat{\gamma }}_{7},{\hat{\gamma }}_{8}\right)}^{T}$.

Figure 6 shows the estimated number of calls from the previous day, the day-of-the-week effects, and the effect from advertising. If we compare the top line of Figure 6 with that of Figure 4, the 3 troughs observed in Figure 4 correspond to periods with little or no advertising. However, the converse was not true for the 2 peaks from late December 2000 to mid-January 2001 and the second half of May 2001, where we observe a sudden increase in daily call volume. This increase was modeled by the varying coefficients and therefore demonstrates the need to incorporate these terms into the models.

Table 1 shows the mean square errors for the 5 different models. The present model had the smallest MSE and fitted best.

## DISCUSSION

Quitline is a major public health intervention program for smoking cessation. It is a media tobacco cessation campaign that is free and available to all members of the general population who have access to a television and a telephone. Although there is convincing evidence for the effectiveness of this program as an initial point of contact for smokers who wish to quit, the underlying relationship between the number of calls to Quitline and the intensity and placement of anti-smoking advertisements is poorly understood.^8^^–^^10^ This can be partly explained by the complexity of unraveling these relationships (as they vary through time) and the effects of other, unknown factors on call volume, as well as by limitations in the statistical methods used in practice.

Although the intensity and placement of anti-smoking advertisements are the primary factors that influence the number of calls to Quitline, a number of other factors may have an impact. These include public relations activity (such as World No Tobacco Day, New Year, and the launch of a new advertisement), residual effects from a previous campaign, and information obtained from other sources. With no available data on these unknowns, there are several advantages of semi-varying models. The nonparametric component of the semi-varying coefficient models incorporates these factors into the modeling by allowing call volume to vary smoothly with time. Incorporation of this term seems to permit detection of the underlying structure, given that there is no real evidence of a long-term trend. In this modeling framework, in addition to fixed day-of-the-week effects, we expect the day effects to vary and to depend on the number of calls received on the previous day.

Very few studies have focused on modeling the relationship between the intensity of anti-smoking advertising associated with the Quit campaign, placement of anti-smoking advertisements, and call volume to Quitline. In fact, to our knowledge, only 2 studies implemented a regression-type modeling strategy to monitor trends in call volume.^8^^,^^15^ Our findings are in agreement with both of these previous studies, which found an association between the TARP values and calls to Quitline.

The fixed-effects regression approach implemented in Miller et al^8^ does not adequately model the underlying non-linear trend in call volume. Although the semi-parametric approach in Erbas et al^15^ extends the classical regression modeling framework by modeling the time trend nonparametrically, the models do not reflect the stochastic nature of the trend. Semi-varying coefficient models, although new, are useful in modeling data, such as call volume, where there is little or no information on other factors related to the at-risk population—in this case, the “at-risk” population of smokers. The varying coefficient approach is a useful technique for modeling data with a strong underlying trend component. There are a number of strengths to this analytical approach. In contrast to the common fixed effects regression techniques used to model trends in call volume and its association with fixed covariates, varying coefficient models allow the underlying time trend to depend on fixed covariates that also vary with time, thereby explaining more of the variation in call volume attributed to other unknown or unstudied factors. We acknowledge some limitations that should be considered when using this method. Although useful for analyzing complex data with an underlying trend, the methods tend to “break down” when analyzing data with many zeros and outcomes of rare events. Nevertheless, the methods are very useful for analyzing large-scale population-based public health data.

Pooling the data into days, as in this study, allows us to establish a fixed relationship between daily call volumes and advertising, in addition to the day-of-the-week effects. However, to fully understand the impact of anti-smoking advertisements we must consider additional covariates, such as level of audience involvement in particular television shows, replacement of advertisements by time of day, and lag effects from exposure to the advertisements. To do this, we need to examine hourly data. Modeling hourly data is a challenge methodologically, as zero counts are frequent both in the outcome variable and potential covariates. A number of models are possible, for example, incorporating a nonparametric component to a zero-inflated Poisson regression model,^16^ which will be considered in future research in this area.
